# Design of multi-functional 2D open-shell organic networks with mechanically controllable properties†
†Electronic supplementary information (ESI) available: (1) plot of twist angle *vs*. energy profiles for TPM and PTM molecules, (2) plot of bi-axial *vs*. uni-axial strain, (3) plots of structure and spin density for PTM 2D-COF upon strain, (4) plots of band structure of PTM 2D-COF upon strain, (5) plot of SOMO–SUMO energy difference for TPM and PTM molecules, (6) full details of magnetic coupling calculations. See DOI: 10.1039/c6sc01412g



**DOI:** 10.1039/c6sc01412g

**Published:** 2016-08-31

**Authors:** Isaac Alcón, Daniel Reta, Iberio de P. R. Moreira, Stefan T. Bromley

**Affiliations:** a Institut de Química Teòrica i Computacional de la Universitat de Barcelona (IQTC-UB) , Departament de Ciència de Materiales i Química Física de la Universitat de Barcelona , C/Martí I Franqués 1 , 08028 Barcelona , Spain . Email: s.bromley@ub.edu; b Institució Catalana de Recerca i Estudis Avançats (ICREA) , 08010 Barcelona , Spain

## Abstract

Triarylmethyls (TAMs) are prominent highly attractive open shell organic molecular building blocks for materials science, having been used in breakthrough syntheses of organic magnetic polymers and metal organic frameworks. With their radical π-conjugated nature and a proven capacity to possess high stability *via* suitable chemical design, TAMs display a variety of desirable characteristics which can be exploited for a wide range of applications. Due to their particular molecular and electronic structure, the spin localization in TAMs almost entirely depends on the dihedral angles of their three aryl rings with respect to the central methyl carbon atom plane, which opens up the possibility of controlling their fundamental properties by twisting the three aryl rings. Aryl ring twist angles can be tuned to a single value by specific chemical functionalisation but controlling them by external means in organic materials or devices represents a challenging task which has not yet been experimentally achieved. Herein, through rational chemical design we propose two 2D covalent organic frameworks (2D-COFs) based on specific TAM building blocks. By employing *ab initio* computational modeling we demonstrate that it is possible to externally manipulate the aryl ring twist angles in these 2D-linked TAM frameworks by external mechanical means. Furthermore, we show this structural manipulation allows for finely tuning the most important characteristics of these materials such as spin localization, optical electronic transitions and magnetic interactions. Due to the enormous technological potential offered by this new class of material and the fact that our work is guided by real advances in organic materials synthesis, we believe that our predictions will inspire the experimental realization of radical-2D-COFs with externally controllable characteristics.

## Abstract 2

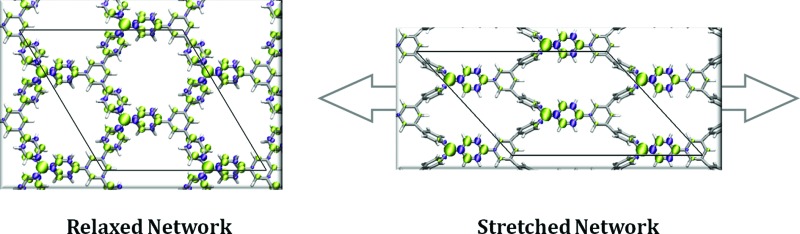
Controlling spin localization, structure, electronic energy levels and magnetic interactions in a flexible open-shell 2D organic framework by external mechanical strain.

## Introduction

**Fig. 1:**
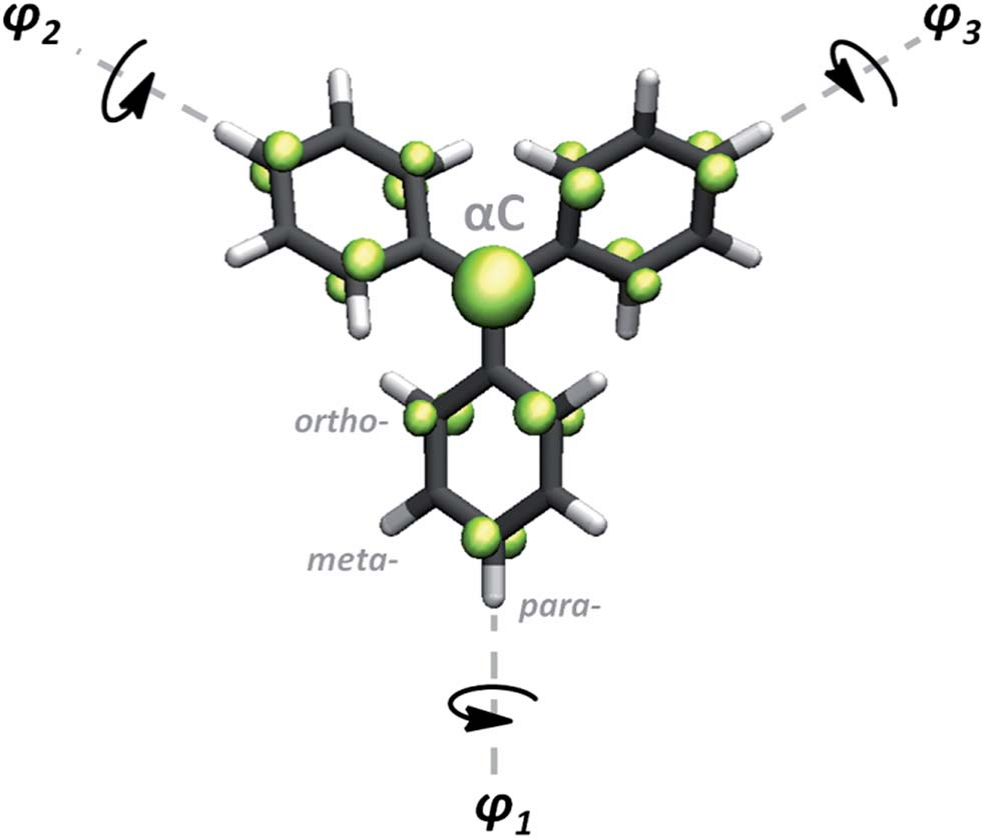
Generic molecular structure of TAMs. *φ*_1_, *φ*_2_ and *φ*_3_ represent the aryl ring twist angles mainly determining the spin density distribution associated with the unpaired electron (green) in TAMs.^11^

Since their discovery by Moses Gomberg in 1900,^1^ triarylmethyls (TAMs) have become one of the most prominent classes of molecules in the field of organic radical chemistry.^2,3^ All TAMs are composed of three aryl rings bonded to a central methyl carbon atom (αC) where their unpaired electron mainly resides (Fig. 1). Due to the steric protection provided by the three aryl rings, TAMs display high radical stability which has allowed chemists to synthesize more than hundred TAM derivatives that have been used for different applications and as building blocks for multi-functional materials and devices.^4,5^ The radical π-conjugation in TAMs is responsible for many of their key characteristics such as fluorescence,^6^ enhanced electrical conductivities,^7^ redox-activity^8,9^ and magnetoresistance phenomena.^10^ As a consequence, TAMs have been utilized for the preparation of organic magnets,^12–14^ non-volatile memory devices,^8^ molecular wires,^10,15^ switchable surfaces,^9^ dynamic-nuclear polarization components,^16^ organic light emitting diodes^17^ and nano-porous magnetic sensors,^18^ among others.^19^

Recently, we used *ab initio* computational modelling to show that, due to the π-conjugated nature of the unpaired electron in TAMs, the degree of spin delocalization in this type of molecule almost entirely depends on the dihedral angle of each aryl ring with respect to the central methyl carbon atom plane (*φ*_1_, *φ*_2_, *φ*_3_ in Fig. 1).^11^ Based on π-overlap arguments,^20–23^ we found that a linear relationship exists between the spin density on αC and the average of the cosine squared of the three dihedrals (cos^2^ *φ* = (cos^2^ *φ*_1_ + cos^2^ *φ*_2_ + cos^2^ *φ*_3_)/3). Moreover, we found that this relationship is independent of both the chemical functionalization of the considered TAM derivative and the system's temperature. In TAMs, the presence of the unpaired electron is responsible for their most interesting properties. Hence, due to the spin-localization structural relationship in these radicals, the manipulation of aryl ring twist angles represents a potentially powerful tool for controlling these properties in a precise manner.

Aryl ring twist angles in π-conjugated molecules are mainly determined by their chemical functionalisation through the correspondingly induced steric hindrance.^24,25^ Some works studying bi-phenyl molecular junctions have pointed to the possibility of using electric fields^26^ or electro-chemical potentials^27^ to externally manipulate dihedral angles within molecular devices. However, chemical design is, by far, the most utilized approach to finely tune this structural parameter to a certain value in order to determine the associated electrical,^24,28^ magnetic,^29^ optical^30^ and electro-chemical properties.^25^ The challenging task of externally tuning aryl ring twist angles in single-molecule devices comes from the extreme difficulties associated with: (i) controlling the orientation of the molecular units within the device^15^ and, (ii) the need to direct the external stimulus uniquely to twist aryl rings without causing other physico-chemical phenomena which may also interfere with the output characteristics of the system.^10^

Recently, it has been experimentally demonstrated that applying a uniaxial strain onto graphene mono- and multi-layers greatly influences the electrical properties of the 2D material due to the perturbation of its honey-comb like structure.^31–37^ In a very similar way, externally applied strain onto a suitably designed TAM-based material could induce an aryl ring twist within every TAM unit composing the material's skeleton which, in turn, should open the possibility of externally controlling its most fundamental characteristics.^11^ External manipulation of aryl ring twist angles in π-conjugated materials though, as far as we know, has not yet been experimentally achieved, highlighting the challenging task that it represents. To accomplish this objective there are some important criteria regarding the structural characteristics of the TAM-based material to be designed:

(1) The main skeleton of the material must be composed of TAM building blocks in such a way that an external perturbation of the material's structure will induce a significant change of the conformation of the TAM building units composing it.

(2) The bonding that sustains the basic structure of the material must be strong enough to withstand the mechanical loads required to overcome the sterical hindrance between aryl rings within TAM units.

(3) The material's skeleton should be ordered as to ensure a homogeneous structural response when applying the external mechanical stimulus, leading to correspondingly smooth, sensitive and reproducible observable physico-chemical changes.

**Fig. 2:**
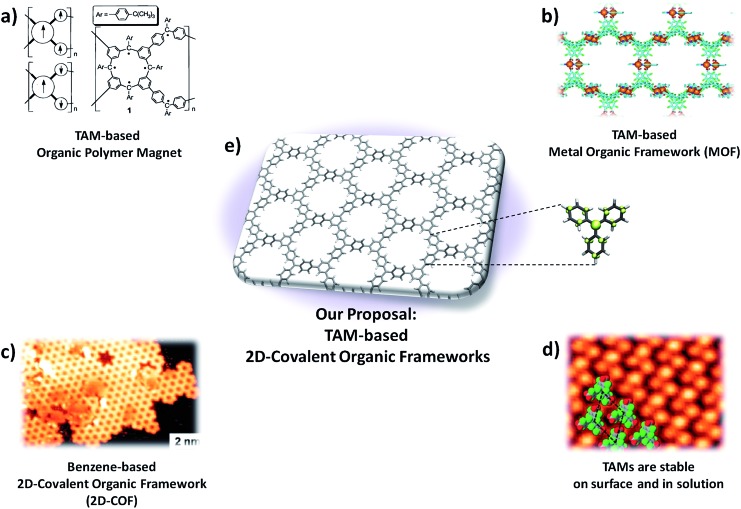
(a) Organic polymer magnet based on the triphenylmethyl (TPM) reported in 2001.^13^ (b) Metal–organic framework based on a perchloro-triarylmethyl (PTM) reported on 2003.^18^ (c) STM image of a 2D-covalent organic framework (2D-COF) formed by the on surface self-reaction of 1,3,5-tri-iodo-benzene.^77^ (d) STM image of a tri-carboxylic PTM monolayer on copper.^89^ (e) TAM-based 2D-covalent organic framework proposed in this study.

Among the different TAM-based functional materials reported to date there are two examples that come close to fulfilling our three criteria. The first is the organic polymer magnet based on a triphenylmethyl derivative (*i.e.* fully hydrogenated TAMs herein abbreviated as TPMs) reported by Rajca *et al.* in 2001 (Fig. 2a).^13^ In this case, the material's skeleton is composed of TPM units which are covalently bonded (criteria 1 and 2) but, as being a 1D organic polymer prepared in solution, its structure consists of a disordered organic network. As a consequence, the application of any type of external mechanical load would tend to generate different types of uncontrolled inelastic structural changes, preventing thus the use of aryl ring twist angles to uniquely determine the characteristics of the stretched material. The second interesting example is a metal–organic framework (MOF) based on a perchloro-triarylmethyl (*i.e.* fully chlorinated TAMs, herein abbreviated as PTMs) reported by Maspoch *et al.* in 2003 (Fig. 2b).^18^ In this case the PTM units also make up the main structure of the material which, in turn, possesses crystalline ordering (criteria 1 and 3). However, the bonding interactions between building blocks are of a weak electrostatic nature and, thus, the strain release would probably change the relative positions of PTM units within the framework before perturbing their inner molecular conformation (*i.e.* aryl ring twist angles), thus also precluding the use of this interesting material for our goal.

Over the last few years a new type of organic material which could realize the proposed three requirements has emerged: so called 2D-covalent organic frameworks^38–41^ (or 2D-COFs). 2D-COFs are organic bi-dimensional layers where initially discrete molecular building blocks occupy ordered positions within a covalent framework (Fig. 2c). The final properties of 2D-COFs entirely depend on the constituent molecular units, their connectivity (*i.e.* how building blocks are covalently bonded) and the utilized methodology to prepare them (preparation can be performed either in solution^42,43^ or on surface^44,45^). 2D-COFs may be understood as a class of material which bridges between organic polymers and MOFs (2a and b), and which possesses the appropriate characteristics for our purpose: *i.e.* covalent bonding and crystalline ordering. A few computational studies have demonstrated the unique characteristics of polyradical 2D-COFs^46–52^ but, due to the inherent instability of open shell molecules, as far as we know, no 2D-COF whose structure is composed of radical building blocks has yet been experimentally reported. However, the demonstrated chemical persistence of TAMs both in solution^53^ and on surfaces^54,55^ (Fig. 2d), the use of TAMs as building blocks for other types of materials^13,18^ (Fig. 2a and b) and the impressive recent developments in the field of 2D-COFs^40,56^ represent strong support in favour of the possible preparation of TAM-based 2D-COFs in the near future (Fig. 2e).

In this study we have rationally designed two different 2D-COFs based on the triphenylmethyl (TPM 2D-COF) and the perchloro-triarylmethyl (PTM 2D-COF), which represent the most important families of TAM derivatives for materials science to date. By means of density functional theory (DFT) calculations we have optimized the corresponding 2D periodic model structures and studied their resulting physico-chemical characteristics. Our results show that both proposed 2D-COFs are stable planar nanoporous materials consisting of ordered arrays of linked open-shell centres. Application of a uniaxial strain to both networks gives rise to a smooth perturbation of the molecular structure within every TAM unit composing the material. In agreement with our previous study on single molecule TAMs,^11^ the strain-induced aryl ring twist gives rise to a monotonic and homogeneous change in the spin localization within every radical centre. Accordingly, the electronic and magnetic properties of the materials are also found to change, demonstrating the validity of our approach to design 2D multi-functional materials with externally controllable characteristics. Further, we have carried out *ab initio* molecular dynamics (AIMD) simulations to test the effect of finite temperatures on the studied properties of the 2D networks. Here we observe a random small deviation in the studied variables produced by thermally activated bond vibrations. However, as we observed for isolated TAM monomers, the average value of each variable over time is clearly determined by the corresponding aryl ring twist angles. By comparing the different behaviours at finite temperatures of the differently functionalized 2D-COFs (TPM and PTM based) we also demonstrate the essential role of chemical functionality in determining the controllability of the material's properties by external manipulation of aryl ring twist angles.

## Methodology

All the 2D model structures of the 2D-COFs considered in this work were constructed using periodic boundary conditions and fully optimized using an efficient cascade methodology. The universal force field (UFF)^57*a*^ was first used within the General Utility Lattice Program (GULP)^57*b*^ to obtain pre-optimized structures (atomic positions and cell-parameters). This was followed by a second full optimization of both structure and cell parameters with DFT using the PBE functional^58^ and a light numerical basis set. Another full DFT optimization was performed using the hybrid PBE0 functional^59^ and light numerical basis set. Finally, single point calculations using PBE0 hybrid functional and tight numerical basis set were performed to obtain the electronic structure and properties of the system. Due to the large cell size (*a* = *b* = 21.6 Å with *c* = 40 Å to provide a large vacuum separation between repeated sheets in the *c*-direction) all periodic DFT calculations were performed at the gamma point in reciprocal space.

To mimic the application of a uniaxial strain on our 2D materials, we performed a series of restricted optimizations (following the cascade methodology indicated above), systematically increasing one of the in-plane cell parameters (by 0–5 Å) allowing the other in-plane cell parameter and internal atomic positions to relax. We note that due to the symmetry of the considered 2D-COF, increasing either the *a* or *b* cell parameter is equivalent. Biaxial isotropic strain was also investigated by applying the same degree of distortion to both *a* and *b* cell parameters simultaneously and optimizing all the internal independent coordinates. This kind of isotropic distortion was found to directly affect the sigma-bonded skeleton and the energy variation was significantly larger than the separate *a* (or *b*) uniaxial strain, even for small distortions. Results for biaxial strain are given in the ESI.† Herein we will focus our study on the uniaxial distorted structures.

The thermally-activated response of the materials was modelled *via ab initio* molecular dynamics (AIMD) simulations. Calculations were run for 5 ps (1 ps of equilibration plus 4 ps of production) at 300 K with fixed lattice parameters using the Bussi–Donadio–Parrinello^60^ thermostat and the hybrid PBE0 functional.

All the above described DFT-based calculations were performed using the FHI-AIMS code,^61,62^ employing the ferromagnetic solution of the system. All reported atomically partitioned spin populations were calculated using the Hirshfeld method.^63^

For the calculation of the relevant (*i.e.*: non zero) magnetic coupling constants, we used the CRYSTAL09 code^64,65^ and the B3LYP^66^ hybrid density functional based method with a standard 6-21G* basis set for all atoms.^67,68^ ITOL values have been fixed to 7,7,7,7,14 to force stringent numerical convergence of energy and gradients and a shrinking factor of 6 within the Monkhorst–Pack scheme to define the reciprocal space mesh in the 2D irreducible Brillouin zone leading to a set of 20 *k*-points.

The description of the magnetic properties of a system of interacting localised magnetic moments is based on the Heisenberg–Dirac–Van Vleck (HDVV) spin Hamiltonian^69^
1
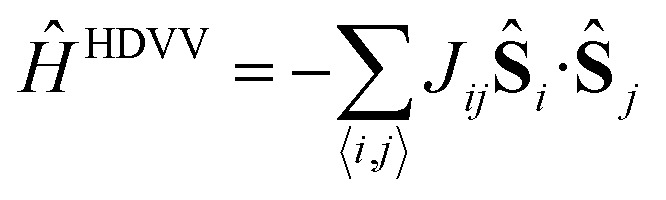
where *J*_*ij*_ is the exchange coupling constant between the **ŝ**_*i*_ and **ŝ**_*j*_ localized spin moments and the *i*, *j* symbol indicates that the sum is over nearest neighbours only. A positive value of the exchange coupling constant *J*_*ij*_ corresponds to ferromagnetic (FM) interactions, while negative values describe an antiferromagnetic (AFM) interaction (parallel and antiparallel spins respectively). The number, sign and magnitude of the most relevant *J*_*ij*_ determine the low-energy spectrum of the system and, consequently, the magnetic structure of the system. The extraction of the different *J*_*ij*_ values is based on the mapping approach described elsewhere.^70^ In this approach, the extraction of the relevant magnetic coupling constants of the system is based on the use of broken symmetry solutions to represent different magnetic solutions of the Ising spin Hamiltonian^69^
2
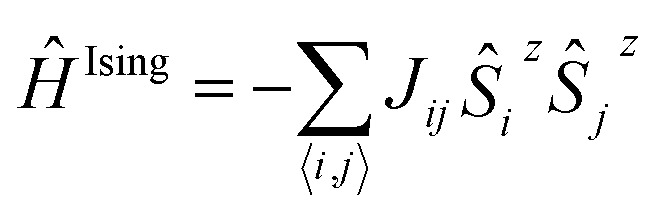
where *J*_*ij*_ are the exchange coupling constants and the ŝ_*i*_^*z*^ and ŝ_*j*_^*z*^ are the *z* components of the corresponding **ŝ**_*i*_ and **ŝ**_*j*_ localized spin moments. The Ising Hamiltonian corresponds to the diagonal terms of the HDVV Hamiltonian. It has been shown that a mapping procedure between the energy values of different broken symmetry solutions and the corresponding expectation values of the HDVV Hamiltonian can be related by using the Ising energy expectation values. A detailed description of this procedure can be found in ref. 71 and a general discussion of the mapping approach to extract *J* values from BS solutions in related systems can be found in.^70,72,73^ In the ESI† we provide the relevant broken symmetry solutions and the particular relations to extract the non-zero magnetic coupling constants described below.

## Results and discussion

The most important parameters that determine spin localization in TAMs are the twist angles of the three aryl rings with respect to the bonding plane of the central carbon atom (see Fig. 1).^11^ In this respect, the role of chemical functionalization seems to be limited to determining the aryl ring twist angles due to steric hindrance, but other effects such as the electro-donating/withdrawing nature of substituents play a secondary role. The same steric repulsion, as determined by chemical functionality, also determines the rotational freedom of the three aryl rings and thus the ease with which their twist angles may be externally manipulated. Therefore, to explore the role of chemical functionality on the controllability of the material's properties following our approach we carried out our study on two differently functionalized 2D-COFs constructed separately with the TPM and PTM TAM derivatives. These two TAM derivatives present different degrees of aryl ring steric hindrance, due to the different functionalization with H (low) and Cl atoms (high), respectively. PTMs, in particular, have outstanding radical stability^53^ due to the “shielding” effect provided by chlorine atoms but, in turn, manipulation of its aryl rings twist should be much more difficult in comparison with the fully hydrogenated TPM, due to the associated higher steric constrain (see the twisting energy profiles for each molecule in the ESI†).

### Material design

**Fig. 3:**
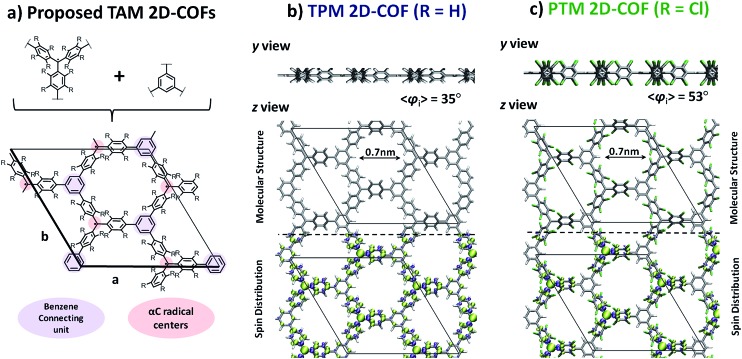
(a) TAM and benzene building blocks (up) utilized to construct the TAM 2D-COF periodic structure proposed in this work (down). The indicated *a* and *b* in-plane cell parameters are equivalent. Optimized structures of the TPM (b) and PTM (c) 2D-COFs. The spin densities are also shown in the bottom area (alpha = green, beta = purple) of the *z*-view. Lateral *y*-view is provided (top) for facilitating the comparison between the different aryl ring twists within each network. Atom colour key: C – grey, H – white, Cl – green.

In the design process our first objective was to ensure that the 2D networks maintained both their planarity and a multi-radical character. To avoid electron pairing (*i.e.* preservation of multi-radical character) based on topological considerations of molecular orbital principles,^74–76^ the radical centres should be connected *via meta*-positions with respect to each other (*i.e.* in a non-Kekulé fashion). However, due to the propeller-like structure of TAMs, connections through the *meta*-positions of aryl rings could lead to non-planar or highly constrained networks. Thus, to ensure planarity, the 2D networks should be propagated through the *para*-positions of TAMs' aryl rings (see Fig. 1 for position labelling). To overcome such contradictory requirements we introduced the use of benzene rings (commonly utilized building blocks composing many reported 2D-COFs^77^) that would connect three TAM units in *meta*-to-*meta* fashion one respect each other while being covalently bonded through the *para*-positions of the TAM aryl rings. Thus, in our resulting proposed periodic structure depicted in Fig. 3a (composed of three TAM units – shaded red, and three connecting benzene rings – shaded purple) both the multi-radical character of the networks and their planarity should be guaranteed.

Based on our designed structure (3a) we constructed two different networks with R = H (TPM 2D-COF) and R = Cl (PTM 2D-COF) whose atom coordinates and cell parameters were fully optimized (see Methodology section). We note that both our networks were constructed with all TAM units having the same helicity of their propeller-like structure; *i.e.* with all aryl rings within the unit cell twisted in the same direction. Experimentally, it is possible that networks may be obtained with some TAM centres possessing different helicities (*i.e.* plus and minus). However, for the properties studied in this work we do not expect this small conformal variability to significantly affect our predictions. As shown in Fig. 3b and c, after optimization, both the TPM and PTM 2D-COFs become, effectively, planar networks with stable radical centres placed in ordered positions within the 2D nano-porous array (pore radius: 0.7 nm). Benzene rings and αC radical centres (purple and red coloured in Fig. 3a, respectively) lay parallel to the network plane, whereas the corresponding aryl rings are partially twisted by 35° and 53° for the TPM and PTM 2D-COFs, respectively (see *y* view in Fig. 3b and c top). These angle values are slightly higher than those ones for the corresponding single molecules (34° and 49°, respectively), which may be attributed to the slightly higher steric congestion within the 2D materials (especially for the PTM case).

Examining the spin distribution (see Fig. 3b and c bottom), it can be seen that all TAM units within both 2D-COFs present spin density (alpha = green, beta = purple) which is mainly located on every αC with very little, or no spin population on the connecting benzene rings. Moreover, the TAM units are, in both cases, ferromagnetically coupled (*i.e.* all unpaired electrons present the same spin alignment). These two features are characteristic of non-Kekulé structures which, besides avoiding electron–electron pairing (*i.e.* quinoidization), induce FM spin alignments.^78^ The FM solutions lay 16.2 and 1.6 meV (per unit cell) below the first excited AFM solutions for the TPM and PTM 2D-COFs, respectively. The lower FM stability for the PTM 2D-COF may be associated with a weaker interaction between its unpaired electrons due to the much more pronounced twisted conformation of its aryl rings (*φ*_*i*_ = 53°) and the subsequent suppression of spin density on the connecting benzene units. We also note that the closed-shell solutions for the TPM and PTM 2D-COFs are 1.5 and 2.2 eV higher (per unit cell) than the corresponding open shell FM solutions.

The obtained bandgap values of 2.17 and 2.86 eV for the TPM and PTM 2D-COFs, respectively, are indicative of a localization of the electronic states near the Fermi energy level. This suggests that unpaired electrons are not delocalized through the whole π-conjugated electronic system, but rather confined within each corresponding TAM unit. Therefore, our designed TPM and PTM 2D-COFs are not in-plane electrical conductors as other 2D covalent π-conjugated organic materials (such as graphene, where a local perturbation has an effect on the whole π electronic system) but, more likely, covalent frameworks composed of electronically isolated TAM radical centres. However, due to the electro-active nature of TAMs already experimentally exploited for the preparation of non-volatile memory devices,^8^ these TAM-based 2D-COFs may be potentially used as flexible data storage materials where every TAM centre within the ordered array may behave as a logic bit with two or more redox states and different output signals.

### Structure response to strain

The application of a uniaxial strain onto mono- and multi-layered graphene has been experimentally proven to be a very effective way to tune the electrical properties of 2D materials.^31–37^ Although several theoretical works have demonstrated the potential of applying a bi-axial strain to tune fundamental characteristics of many 2D extended systems^79–81^ (including graphene^82^ and certain 2D-COFs^83^) in experiments monolayered materials are often uniaxially stretched between two electrodes.^32^ As mentioned above, we focus on uniaxial strain by increasing the *a* (or, equivalently, *b*) cell parameter of our 2D-COFs as it was found to be the energetically most facile way of distorting the material and was that which induced a high degree of aryl ring twisting, which is the principal objective of our work. We compare this situation with that for bi-axial strain in the ESI.†

**Fig. 4:**
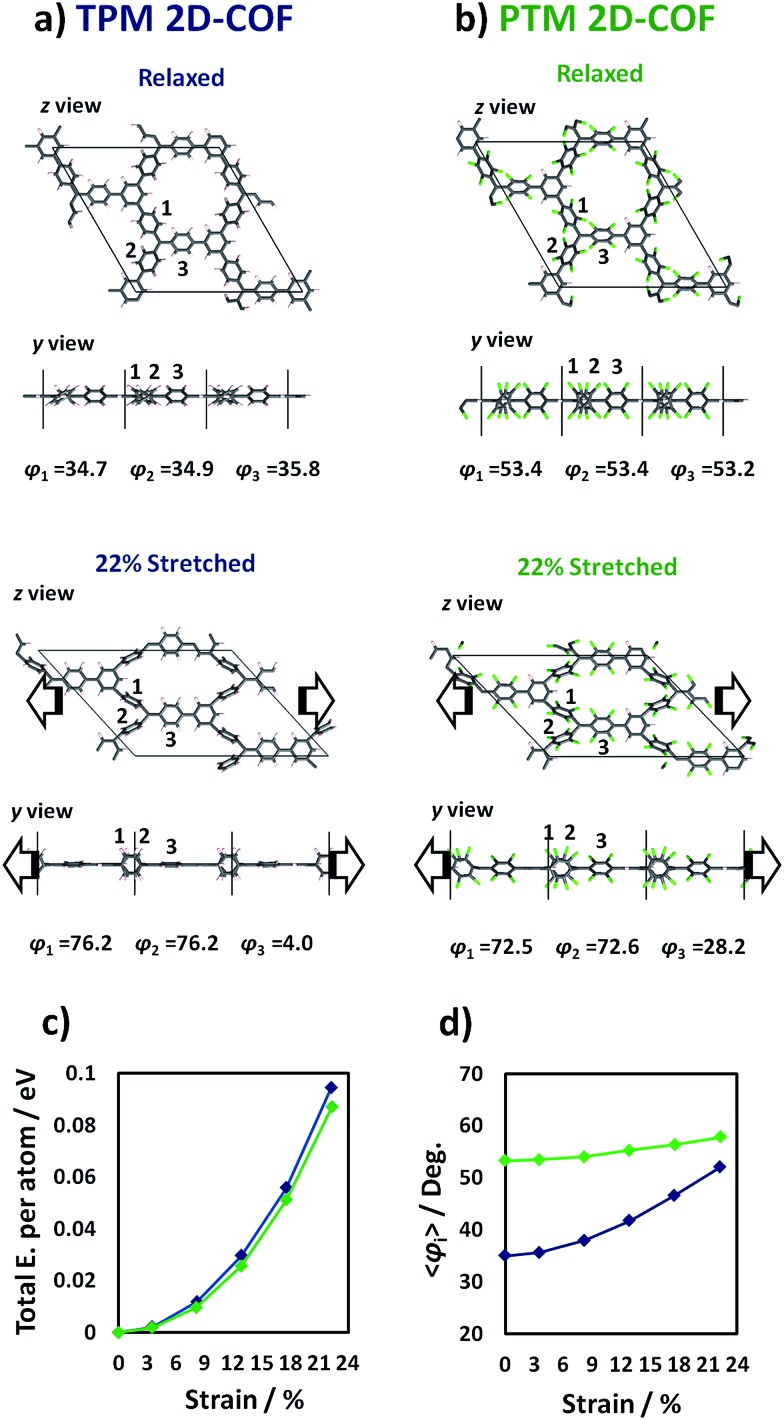
Out-of-plane (*z* view) and in-plane (*y* view) perspectives of the relaxed (up) and 22% stretched (down) TPM (a) and PTM (b) 2D-COFs. Both nets were stretched from 0 to 5 Å in the *x* direction parallel to the network plane (see arrows) while allowing the periodic cell to relax in the other in-plane direction (*y*). The twist angles of the indicated aryl rings (1, 2 and 3) are provided below each structure. (c) Total energy variation per atom (relative to the relaxed structure) *vs.* applied strain for the TPM (blue) and PTM 2D-COFs (green). (d) Variation of *φ*_*i*_ (where *φ*_*i*_ = (∑*φ*_*i*_)/9 and *φ*_*i*_ are the aryl ring twist angles with respect to the corresponding αC plane) *vs.* applied strain for the TPM (blue) and PTM 2D-COFs (green).

Fig. 4 Fig. 4 shows the *z* and *y* views of the relaxed structures (top) and most stretched (+22%) structures (bottom) for the TPM (4a) and PTM (4b) 2D-COFs. As it can be observed, uniaxially stretching the networks effectively generates a twist on some of the aryl rings of the constituent TAMs. Specifically, for both cases, two of the three aryl rings within each TAM unit twist towards more orthogonal positions (1 and 2 in 4a and b) with respect to the 2D network's plane, whereas the third one becomes more flattened respectively (3 in 4a and b, see twist angle values below each conformer). This twisting effect arises from the compression of the structure of the material in the in-plane direction perpendicular to the applied strain and the subsequent increase in steric hindrance between adjacent aryl rings. In Fig. 4c it can be seen that the energetic cost associated with uniaxially stretching the 2D-COFs by more than +20% of their initial size is rather modest (*ca.* 0.1 eV per atom). Graphene monolayers, for comparison, suffer irreversible ruptures when being uniaxially stretched by +3%.^32,34,37^ Moreover, our calculated Young's modulus for both networks (58 and 55 GPa for TPM and PTM 2D-COFs, respectively, as calculated for in-plane strains covering 0–15%) is around 20 times smaller than that of graphene (1024 GPa),^84^ which corroborates the relatively high elasticity of our designed covalent networks. This property may be understood by their nanoporous structure which offers a number of mechanical relaxation modes (*i.e.* aryl ring bending, twisting, bond lengthening, *etc.*) upon stretching as compared to the perfectly flat and densely tessellated graphene. We also note that, with uniaxial strain, the nanopores of the 2D-COFs become elongated parallel to the stretched direction (1 nm) and narrower with respect to the perpendicular direction (0.3 nm), as compared to the more radially symmetric pores (0.7 nm diameter) of the unstrained structures.

In Fig. 4d we plot the average twist angle of all aryl rings within the unit cell with respect to the corresponding central αC plane (*φ*_*i*_) against uniaxial strain. Here it can be seen that, although not all aryl rings rotate in the same direction (see angle values in 4a and b), their average twist angle smoothly increases during the stretching process for both networks. Moreover, as already expected, the TPM 2D-COF, with a lower steric hindrance imposed by its phenyl rings, presents a wider variation of *φ*_*i*_ (blue curve in 4d) as compared to the more sterically hindered PTM 2D-COF (green curve in 4d).

### Spin localization response to strain

**Fig. 5:**
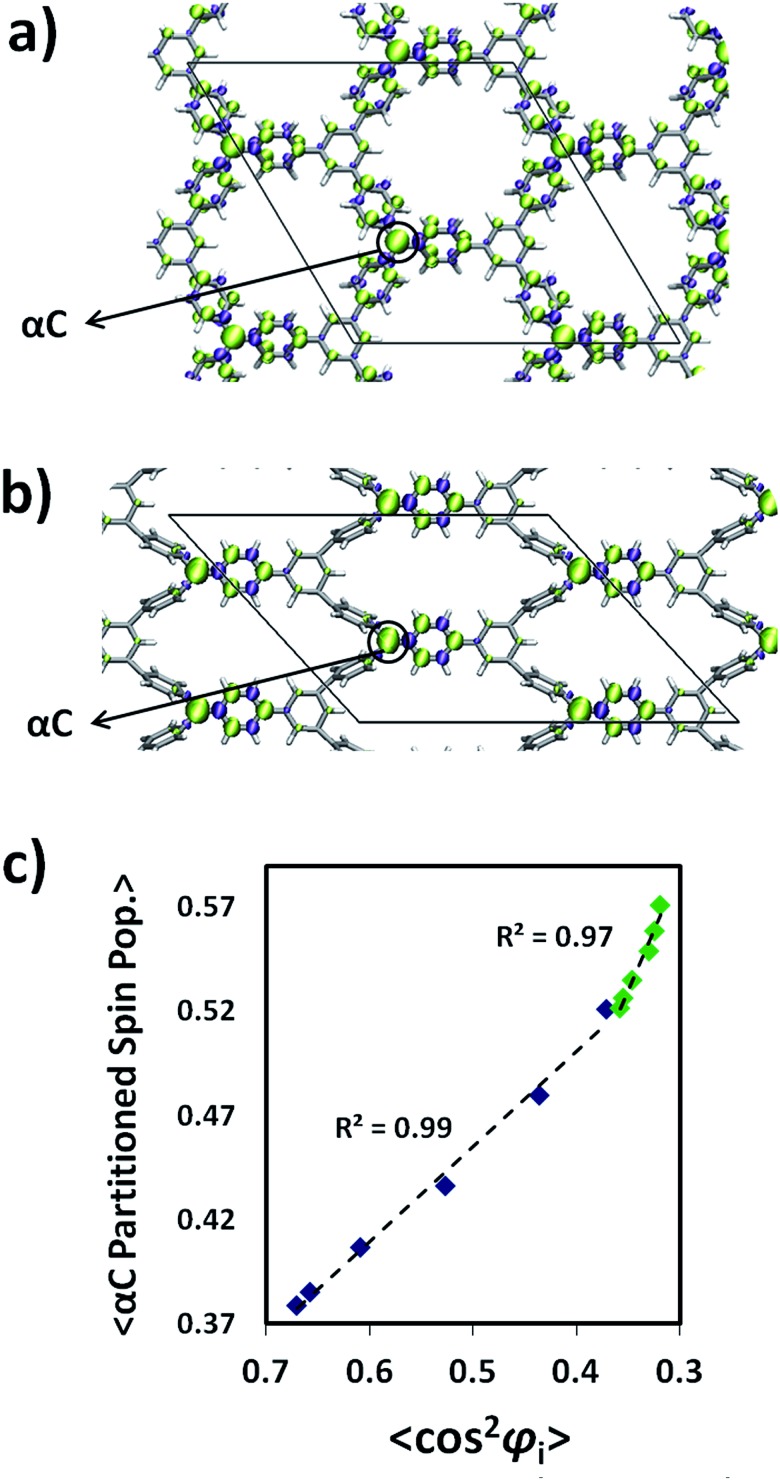
Relaxed (a) and 22% stretched (b) TPM 2D-COF structures with the corresponding represented spin density (alpha = green, beta = purple). (c) Average αC-partitioned spin population against cos^2^ *φ*_*i*_ (where cos^2^ *φ*_*i*_ = (∑cos^2^ *φ*_*i*_)/9 and *φ*_*i*_ are the aryl ring twist angles with respect to the corresponding αC plane) for the differently stretched conformations for the TPM (blue) and PTM (green) 2D-COFs.

The change in *φ*_*i*_ produced by the strain application (Fig. 4d) should give rise to a net change in spin localization within every radical centre within the networks as found for single TAM molecules.^11^ By examining the spin density distribution for the relaxed (Fig. 5a) and most stretched (Fig. 5b) TPM 2D-COF structures we can see how, upon stretching, the spin distribution becomes localized on every αC and on the flattened aryl rings (similar changes are obtained for the PTM 2D-COF, see ESI†). For single molecule TAMs we found that the spin localization, as represented by the αC-partitioned spin population, linearly varies with the average cosine squared of the twist angles of the three aryl rings with respect to the αC plane (*i.e.* cos^2^ *φ*_*i*_ = (cos^2^ *φ*_1_ + cos^2^ *φ*_2_ + cos^2^ *φ*_3_)/3).^11^ To test this relationship in our uniaxially stretched 2D networks we calculated the averaged αC-partitioned spin population for every conformation and the corresponding cos^2^ *φ*_*i*_ value (where cos^2^ *φ*_*i*_ = (∑cos^2^ *φ*_*i*_)/9 and *φ*_*i*_ is the twist angle of each aryl ring with respect to the corresponding αC plane). Fig. 5c shows the variation of the average αC-partitioned spin population against cos^2^ *φ*_*i*_ for the differently stretched TPM and PTM 2D-COFs (blue and green, respectively).

As it can be seen in Fig. 5c, within both 2D-COFs the average αC partitioned spin population is linearly dependent on cos^2^ *φ*_*i*_ for the differently stretched structures. Hence, the spin-localization structural dependence for single molecule TAMs is applicable to our mechanically stretched TAM-based 2D-COFs, confirming the power of aryl ring twist angles to control the localization of unpaired electrons within our designed materials. It is also worth noting the different responses of the two differently functionalized networks. The TPM 2D-COF, which can accommodate wider twisting angles (see Fig. 4d) presents an accordingly larger spin localization variation (blue points in 5c) as compared to the more structurally constrained chlorinated PTM 2D-COF (green points in 5c).

### Electronic response to strain

**Fig. 6:**
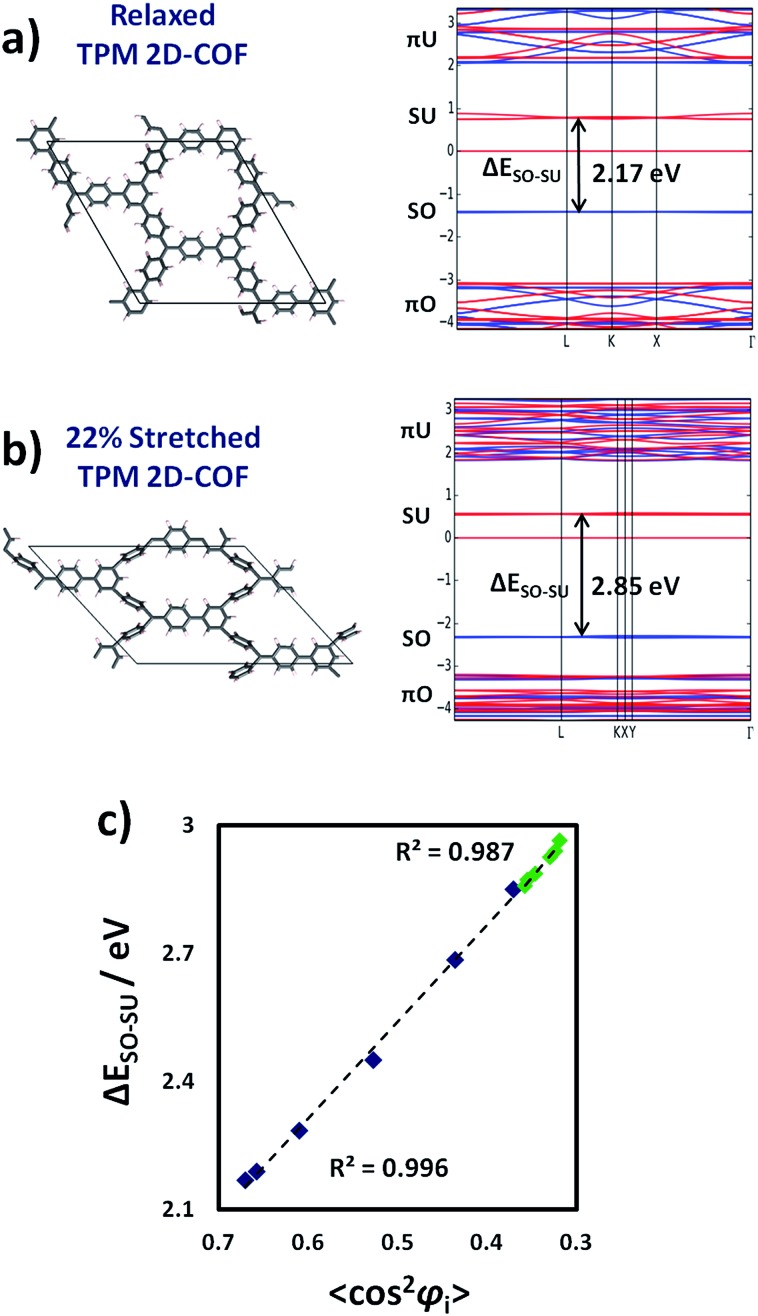
Band structure for the relaxed (a) and 22% stretched (b) TPM 2D-COF. πO/πU and SO/SU correspond to π-double-bond occupied/unoccupied and singly-occupied/unoccupied electronic levels, respectively. (c) Δ*E*_SO–SU_ against cos^2^ *φ*_*i*_ (where cos^2^ *φ*_*i*_ = (∑cos^2^ *φ*_*i*_)/9 and *φ*_*i*_ are the aryl ring twist angles with respect to the corresponding αC plane) for each stretched conformation for the TPM (blue) and PTM (green) 2D-COFs.

The results of Fig. 5 demonstrate that it is possible to localize all unpaired electrons within our designed networks by applying uniaxial strain. As reported for bi-phenyl compounds, localizing electrons in π-conjugated systems dramatically influences their associated energies and, hence, the corresponding optical absorption bands.^30^ Therefore, the mechanically induced spin localization shown in Fig. 5 should be accompanied by significant variations in the electronic band structure of both materials and the related physico-chemical properties. In Fig. 6 the band structure for the relaxed and mostly stretched conformations of the TPM 2D-COF are shown (blue lines = alpha spin, red lines = beta spin). For the PTM 2D-COF we obtained very similar band structure characteristics (see ESI†).

As it can be seen in Fig. 6a, in the relaxed conformation the π-conjugated double-bond system (πO) lays at low energies as a set of bands with non-negligible electronic dispersion, which is in accordance with its intrinsically delocalized nature. Above them and energetically well separated, the singly occupied levels (SO) corresponding to the three unpaired electrons within the unit cell appear as flat electronic bands, suggesting the lack of significant delocalization of these states. The singly unoccupied counterparts (SU) appear at higher energies showing, again, little band dispersion. The flattening of the SO and SU levels are in full accordance with the mostly localized unpaired electrons on αC positions within our networks, as measured by the corresponding spin density distribution (Fig. 5). Above the SO and SU levels, the anti-bonding unoccupied bands of the π-conjugated double-bond system (πU) appear with significant dispersion.

Upon stretching (Fig. 6b), the π-conjugated double-bond bands (πO and πU) become substantially flattened, which indicates that aryl ring twisting cuts the delocalization of the π-conjugated double bond electrons. This effect is in agreement with the experimentally demonstrated electrical conductivity dependence on twist angles in π-conjugated single-molecule devices.^24^ The singly occupied/unoccupied levels (SO and SU) do not present large changes with respect to the shape of their bands, but their relative energy difference (Δ*E*_SO–SU_) increases upon stretching, as indicated by black arrows (Fig. 6a and b). Such type of energy gap variation has also been observed in differently functionalized bi-phenyl compounds where a linear correlation was obtained with the cos^2^ of the corresponding twist angles based, also, on the π-conjugation dependence on the structural parameter.^30^ Therefore, due to the effective localization of unpaired electrons by twisting aryl rings (Fig. 5), we should expect a similar correlation between Δ*E*_SO–SU_ and the average of aryl ring twist angles (cos^2^ *φ*_*i*_) during the uniaxial strain application. In Fig. 6c we plot Δ*E*_SO–SU_ against cos^2^ *φ*_*i*_ (where cos^2^ *φ*_*i*_ = (∑cos^2^ *φ*_*i*_)/9 and *φ*_*i*_ is the twist angle of each aryl ring with respect to the corresponding αC plane) for the differently stretched conformations for both the TPM and PTM 2D-COFs (blue and green, respectively).

As it can be seen in Fig. 6c there exists an excellent correlation between Δ*E*_SO–SU_ and cos^2^ *φ*_*i*_ for the differently stretched TPM (blue) and PTM (green) 2D-COFs. In this case the link between the two parameters seems to be independent on the chemical functionalization of the networks, since for both 2D-COFs Δ*E*_SO–SU_ follows the same correlation line with cos^2^ *φ*_*i*_. We found the same phenomenon when systematically twisting aryl rings in single TAM molecules by constrained optimizations (see ESI†), confirming the generality of this powerful structural–electronic relationship for TAM-based systems. Therefore, the intimate link between the delocalization of electrons and their energies^30^ allows tuning of the electronic band structure of the material by manipulation of aryl ring twist angles (see Fig. 6a and b). In this way, it is possible for instance, to elastically tune Δ*E*_SO–SU_ by up to 0.7 eV within the TPM 2D-COF by simply stretching the network by a 22% of its initial size (blue points in Fig. 6c). We note that these singly occupied/unoccupied levels (SO and SU) participate in a number of important phenomena in TAM-based materials, such as colour absorption bands,^8^ fluorescence^6^ or enhanced single molecule electrical conductivities.^7^ Therefore, the results of Fig. 6 demonstrate that it would be possible to finely tune optical and electrical (*e.g.* sheet to substrate) properties of the material by stretching it in one direction.

### Magnetic response to strain

**Fig. 7:**
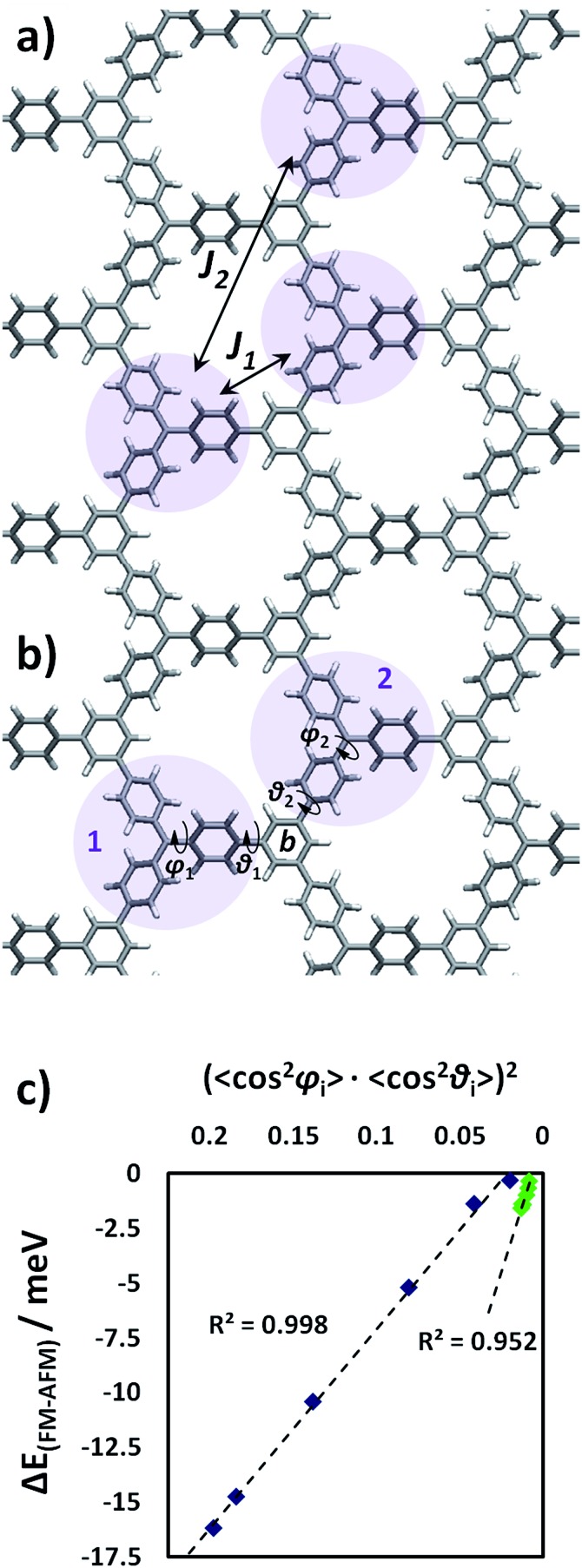
(a) Calculated magnetic coupling constants between first (*J*_1_) and second (*J*_2_) nearest neighbouring TAM centers. Details on the calculation of *J* coupling constants can be found in the Methodology section. (b) Representation of the involved aryl ring twist angles. (c) Energetic difference (meV) between the ground state ferromagnetic solution and the first excited antiferromagnetic one (Δ*E*_FM–AFM_) against (cos^2^ *φ*_*i*_·cos^2^ *θ*_*i*_)^2^ (where *φ*_*i*_ and *θ*_*i*_ are the twist angles of aryl rings with respect to the corresponding αC and benzene ring planes, respectively) for the differently stretched TPM (blue) and PTM (green) 2D-COFs.

As previously mentioned, both the TPM and PTM 2D-COFs present ferromagnetic ordering in their ground state. We calculated the *J* coupling parameters (see Methodology section for computational details and employed equations) between first and second nearest neighbouring radical units (*J*_1_ and *J*_2_ in Fig. 7a). Thus we found that magnetic interactions within second nearest neighbours (*J*_2_ = 0.00074 meV) are negligibly small compared to first neighbours interactions (*J*_1_ = 0.68 meV), which is in concordance with previous studies in organic polyradicals.^78,85^ Fig. 7b depicts a particular pair of first TAM neighbours (1 and 2) whose corresponding unpaired electrons interact with each other on the connecting ring b. The magnitude of the magnetic interaction depends on the degree of delocalization of both unpaired electrons on ring b and, hence, based on pi-overlapping arguments, the magnetic interaction strength between TAM units 1 and 2 can be well estimated by:

3 (cos^2^ *φ*_1_·cos^2^ *θ*_1_) × (cos^2^ *φ*_2_·cos^2^ *θ*_2_)

where each term of the product is associated to the degree of delocalisation of each unpaired electron towards ring b (see Fig. 7b for labels). Due to the homogeneous structural response to the applied strain within both 2D-COFs, instead of treating every magnetic interaction individually, we could embrace the global magnetic response of the material by utilizing the average of the involved aryl ring twist angles. Hence,

4 (cos^2^ *φ*_*i*_·cos^2^ *θ*_*i*_)^2^

should correlate with the ferromagnetic stability of both TAM 2D-COFs, where *φ*_*i*_ and *θ*_*i*_ refer to the twist angles of the *i*th aryl ring with the corresponding αC and benzene ring planes, respectively. Note the overall square in eqn (4), arising from the fact that two unpaired electrons have to be considered in order to properly assess magnetic interactions. For every stretched conformation we extracted the energy difference between the ground state ferromagnetic solution and the first excited anti-ferromagnetic one (Δ*E*_FM–AFM_).

In Fig. 7c we plot Δ*E*_FM–AFM_ against (cos^2^ *φ*_*i*_·cos^2^ *θ*_*i*_)^2^ for the uniaxially stretched TPM and PTM 2D-COFs (blue and green points, respectively). As we can see, Δ*E*_FM–AFM_ linearly varies with (cos^2^ *φ*_*i*_·cos^2^ *θ*_*i*_)^2^ for both TAM 2D-COFs due to the fundamental link between the aryl ring twist angles and the localization of unpaired electrons (Fig. 5). More specifically, as it can be seen in the ESI,† the magnetic coupling constants between first neighbours (*J*_1_) split into two different new magnetic coupling constants upon stretching (*J*_1b_ and *J*_1c_). This is a direct consequence of the uniaxial applied strain and the associated reduction in the symmetry of the triangle relating the three nearest radical centres (see ESI†), which goes from equilateral (relaxed) to isosceles (22% stretched).

More generally, as shown in Fig. 7c, for the most stretched conformations (22% strained) of both materials the ferro- and anti-ferromagnetic solutions become nearly degenerate. This corresponds to a situation that has been mechanically induced where unpaired electrons do not have any energetically preferred spin alignment. Thus the ground ferromagnetic state of both TAM 2D-COFs can be tailored by applying increasing strains, due to the corresponding twisting of aryl rings. Reports of a synthesized tri-TPM derivative, which may be understood as the analogue oligomer of our designed TPM 2D-COF (see ESI† for molecular structure), showed this derivative presents a quartet state (*i.e.* ferromagnetic interactions) at 93 K. Due to the very small obtained *J* coupling constants for both materials though, any spin alignment would be expected only below 10 K (as previously reported for TAM-based magnetic polymers^13^) This would thus limit the potential of our currently proposed materials as organic ferromagnets. However, our results demonstrate the existence of a general, simple and powerful way to externally tune magnetic interactions in multi-radical π-conjugated 2D systems and, hence, we believe these ideas may be applied to other organic ferromagnets, or may inspire the rational design and preparation of similar organic materials with mechanically switchable magnetic properties.

### Effect of finite temperature and the role of chemical functionalization

**Fig. 8:**
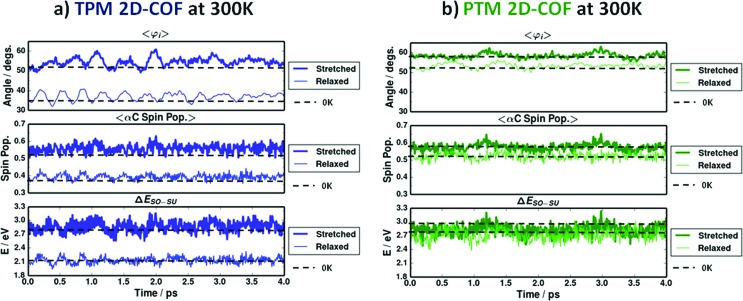
Averaged aryl ring twist angle (up), αC-partitioned spin population (middle) and Δ*E*_SO–SU_ (down; SO and SU are the singly occupied/unoccupied levels corresponding to unpaired electrons) variations during 4 ps of an *ab initio* molecular dynamics simulation (AIMD) at 300 K for 5 ps of time (1 ps of equilibration plus 4 ps of production) for the TPM (a) and PTM (b) 2D-COFs. Dashed black lines represent the corresponding values at 0 K.

As we have seen above, despite the fact that both networks show similar physico-chemical tendencies upon stretching, the range of variation for the TPM 2D-COF characteristics always appears to be much larger than that for the PTM 2D-COF. These differences arise from the correspondingly different rotational freedom of aryl rings, due to the different steric hindrances within each material, as previously explained. Despite being much smaller, the variations in PTM 2D-COF characteristics upon stretching are still detectable, as it can be seen in Fig. 5–7 (green points). However, these results are obtained in the gas phase at 0 K and the effect of finite temperatures, which introduces random variations in the material's properties due to bond vibrations,^11^ might hinder the detection of such mechanically induced physico-chemical changes at realistic conditions. Hence, we probed the behaviour of our designed TAM 2D-COFs under finite temperatures by performing AIMD simulations at 300 K for the relaxed and totally stretched (+22%) conformations of both the TPM and PTM 2D-COFs (see Methodology section for details). Fig. 8 shows the thermal fluctuation of most relevant properties during the simulated 4 ps at 300 K for the relaxed (thin lines) and most stretched (thick lines) conformations of the TPM (a) and PTM (b) 2D-COFs.

In the top panels of both Fig. 8a (TPM 2D-COF) and 8b (PTM 2D-COF) we can see how the average aryl ring twist angle (*i.e.* *φ*_*i*_) vary *versus* time at 300 K. Dashed lines represent the corresponding values at 0 K. Thus, it can be seen that the effect of finite temperatures does not importantly vary the average aryl ring twist angle from the corresponding value at 0 K, but rather introduces a random oscillation around the most stable conformation. Moving to the spin localization (Fig. 8a and b, middle panels) and Δ*E*_SO–SU_ variations (Fig. 8a and b bottom panel) *versus* time we can see that very similar results are obtained. For both quantities the predicted values at 0 K (dashed lines) are well maintained at 300 K and thermal energy simply introduces fast random fluctuations due to thermal vibrations. By comparing the three graphs within each network we can see how the rotational oscillations of aryl rings are approximately followed by the spin localization and the Δ*E*_SO–SU_ fluctuations. This demonstrates that the intimate link between the structural parameter and the physico-chemical properties within both 2D-COFs is well maintained at room temperature. This behaviour was also observed for single molecule TAMs.^11^

However, the most relevant conclusions from Fig. 8 are extracted by comparing the results from the two differently functionalized networks. In the TPM 2D-COF (Fig. 8a) thermal vibrations do not prevent distinguishing the different signals from the relaxed and most stretched conformations, as the perturbation produced by the uniaxial strain is substantially higher than the thermal noise for all studied properties. For the PTM 2D-COF (Fig. 8b) though, the mechanically induced changes on both the spin localization and Δ*E*_SO–SU_ are so small that they are similar in magnitude to the background noise produced by thermal fluctuations (Fig. 8b), hence making their detection over time much more difficult or even not possible. Thus, these results unambiguously demonstrate the key role of chemical functionalization in order to exploit the power of aryl ring twist angles to externally control fundamental characteristics of the materials. On the basis of these results (Fig. 8), the TPM 2D-COF appears to be a better candidate for such a purpose. However, as previously explained, it is more feasible to prepare a PTM 2D-COF in the lab due to the higher stability of the fully chlorinated TAM derivatives. Therefore, an optimal candidate to design a TAM-based 2D-COF in the lab with externally controllable characteristics would be an intermediate derivative (between the TPM and PTM) with the most appropriate balance between chemical stability and structural flexibility. Such TAMs may be found by use of appropriately substituted PTM derivatives^86,87^ or, more generally, in the extensive library of synthesized TAM derivatives.^2^

## Conclusions and outlook

The main objective of this work was to demonstrate that the spin-localization *versus* structural dependence in single-molecule TAMs^11^ could be exploited for the design of multi-functional materials with externally controllable characteristics. The principal barrier for achieving this aim was finding the most appropriate platform to manipulate the aryl ring twist angles by external means; a challenge that, although theoretically proposed,^88^ as far as we are aware has not yet been experimentally achieved for any material. We found 2D-COFs to be the most appropriate platform for such a purpose, due to them realizing a set of three criteria that we believe to be crucial for our proposal. We thus designed two 2D-COFs based on the triphenylmethyl (TPM) and the perchloro-triarylmethyl (PTM), respectively. After 0 K structure optimization using DFT methods, the materials have shown to be stable planar multi-radical nanoporous networks which possess ferromagnetically coupled open-shell centres and moderately large bandgaps.

By mechanically stretching the networks in one of the in-plane directions, we show that most important properties of the designed materials can be finely and elastically tuned. Properties such as spin localization, ferromagnetic interactions and electronic energy levels can all be smoothly controlled by applying a uniaxial strain. This behaviour relies on the manipulation of TAM's aryl rings twist angles upon stretching the organic networks, which is confirmed by the linear dependence that all the studied properties show *versus* the averaged cosines squared of the corresponding dihedral angles.

By performing AIMD simulations at 300 K for the relaxed and most stretched conformations of the two 2D networks, we show that steric hindrance, determined by the chemical functionalization of the networks, plays a crucial role in determining the ease by which aryl rings can be twisted, and thus the external control of materials' properties. PTMs are the most promising building blocks to be used for preparing a TAM-based 2D-COF. However, for better control of properties *via* strain-induced aryl ring twist angles, a TPM-based material would be preferential. To avoid the technical difficulties of preparing a stable material based on TPM derivatives, a specifically designed TAM derivative presenting radical stability and aryl ring structural freedom (*i.e.* between PTM and TPM) would probably be the optimal practical building block for realizing TAM-based 2D-COFs with controllable characteristics by mechanical means. Fortunately, the wide range of existing and ever increasing experimentally synthesised TAM derivatives greatly facilitates finding such a molecular candidate.

In summary, we believe that this work represents the first instance where a material has been specifically and rationally designed to enable control of its most fundamental properties by external manipulation of its aryl ring twist angles. The power of this structural parameter in π-conjugated single molecule systems has been theoretical and experimentally demonstrated.^24,25,28–30^ Our work shows how this fundamental molecular level link between structure and properties can be entirely transferred to extended materials, opening thus its potential use which may give rise to unprecedented technological applications. We hope this work becomes the starting line for the exploration of a new class of functional materials where aryl ring twist angles can be internally tuned (*e.g.* by chemical design) or externally varied (*e.g.* by mechanical strain, electric/magnetic fields, light irradiation) to yield molecular scale control of material's properties. Although of a predictive nature, our work is strongly grounded in the reported synthetic chemistry of both persistent radicals and 2D-COFs. As such we strongly believe in the chemical feasibility of our presented proposal which may inspire the experimental realization of similar extended 2D systems in the close future.
