# Supplementary material for: Design of multi-functional 2D open-shell organic networks with mechanically controllable properties
Source: Chem Sci. 2016 Aug 31;8(2):1027–39. doi: 10.1039/c6sc01412g (PMC5380917; doi:10.1039/c6sc01412g)
Supplement: Supplementary file 1 [file SC-008-C6SC01412G-s001.pdf]

Electronic Supplementary Material (ESI) for Chemical Science.
This journal is © The Royal Society of Chemistry 2016

Electronic Supporting Information:

Design of Multi-functional 2D Open-Shell Organic Networks with Mechanically Controllable Properties

Isaac Alcón[1], Daniel Reta[1], Iberio de P. R. Moreira[1] and Stefan T. Bromley[1,2]*

1 Institut de Química Teòrica i Computacional de la Universitat de Barcelona (IQTC-UB). Departament de Química Física de la Universitat de Barcelona. C/ Martí I Franqués 1, 08028 Barcelona (Spain)

2 Institució Catalana de Recerca i Estudis Avançats (ICREA), 08010 Barcelona, Spain

1. Total energy variation vs. twisting aryl rings in TPM and PTM molecules

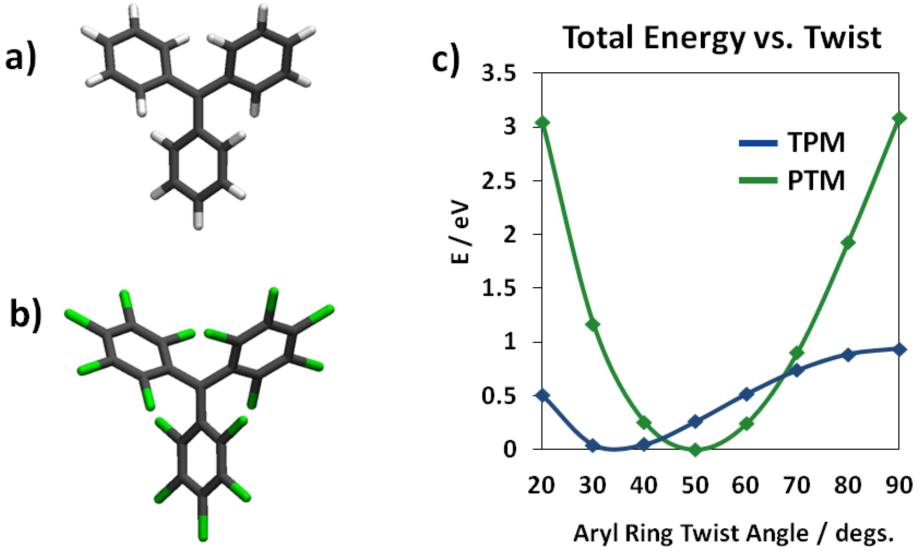

Fig. 1 The three aryl rings of the triphenylmethyl (TPM), (**a**) and perchloro-triarylmethyl (PTM), (**b**) TAM derivatives were systematically twisted from 20 to 90 degrees while relaxing the rest of the molecular structure by constrained optimizations. These calculations were done using the PBE0 hybrid functional[1] and the 6-311++g(d,p) basis set as implemented in the GAUSSIAN09[2] code. c) Resulting total relative energy (with respect to the most stable conformation) variation of the TPM (blue) and PTM (green) molecules against the corresponding $\langle \phi_i \rangle$ value (where $\langle \phi_i \rangle = (\phi_1 + \phi_2 + \phi_3)/3$) during the constrained optimizations.

2. Bi-axial vs. Uni-axial Strain

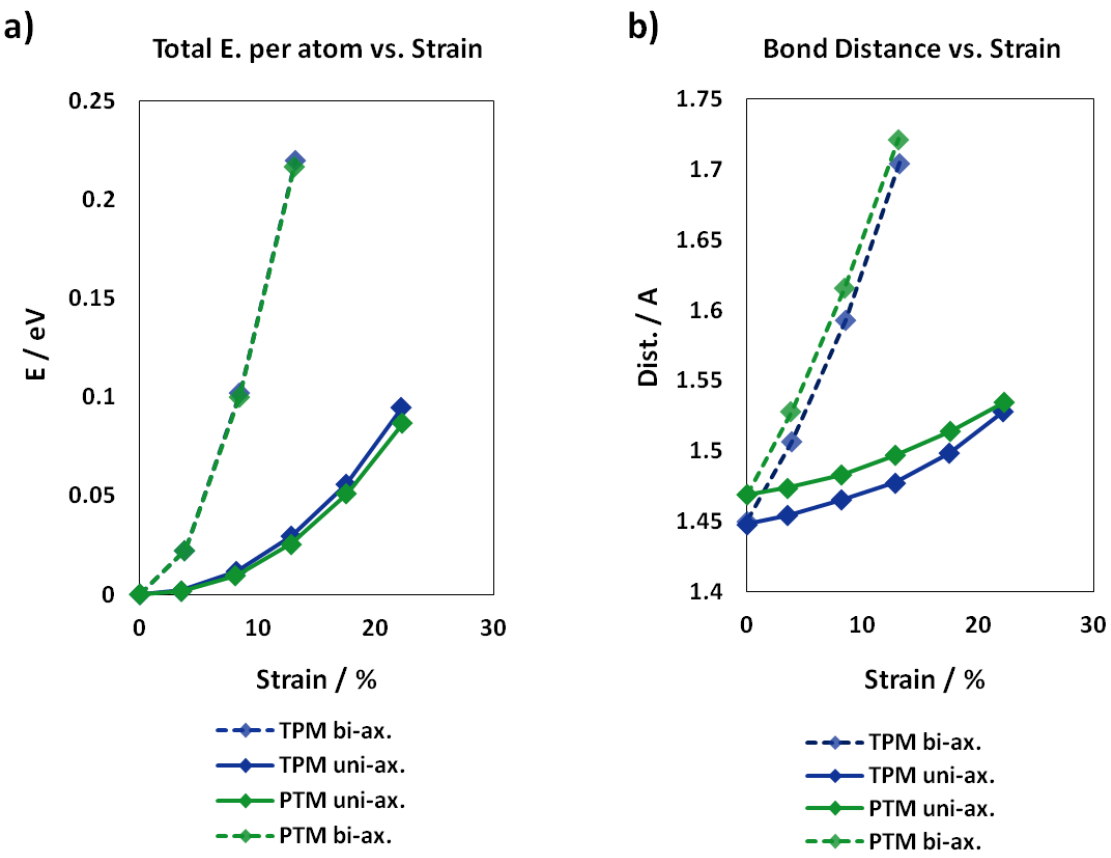

Fig. 2 Total energy per atom (relative to the most stable conformation), (a) and average αC bond distance (b) versus applied strain for both the uni-axial (continuous lines) and bi-axial (dotted lines) stretched TPM (blue) and PTM (green) 2D-COFs.

3. Structure and spin density for PTM 2D-COF

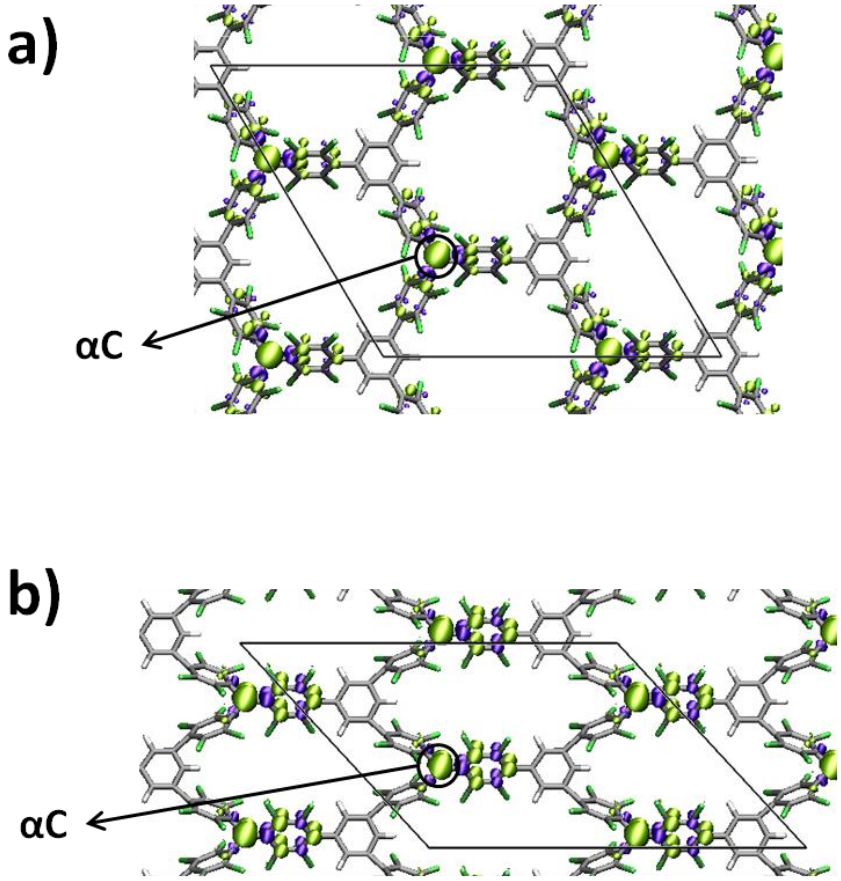

Fig. 3 Relaxed (a) and 22% stretched (b) PTM 2D-COF structures with the corresponding associated spin density (alpha = green, beta = purple). Both the atomic and electronic structures were optimized using the PBE0 hybrid functional[1] within the FHI-AIMS code.[3]

4. Band structures of PTM 2D-COF

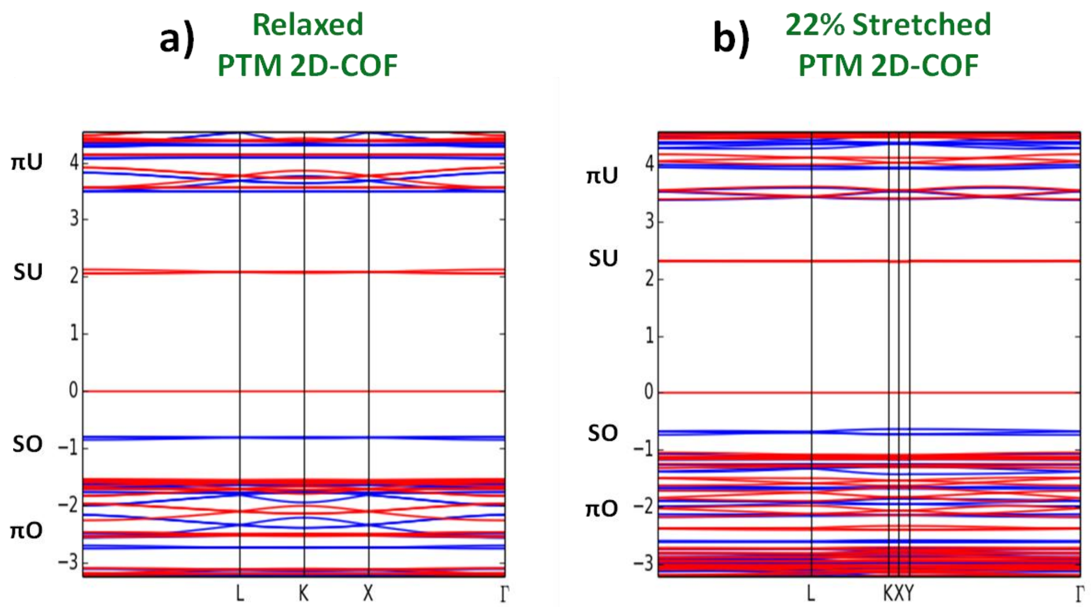

Fig. 4 Band structure for the relaxed (a) and 22% stretched (b) TPM 2D-COF structures. πO, πU, SO and SU correspond to π-double-bond occupied, π-double-bond unoccupied, singly-occupied and singly-unoccupied electronic levels, respectively. Results obtained by using the PBE0 hybrid functional[1] within the FHI-AIMS code.[3]

5. SOMO-SUMO energy difference for PTM and TPM molecules

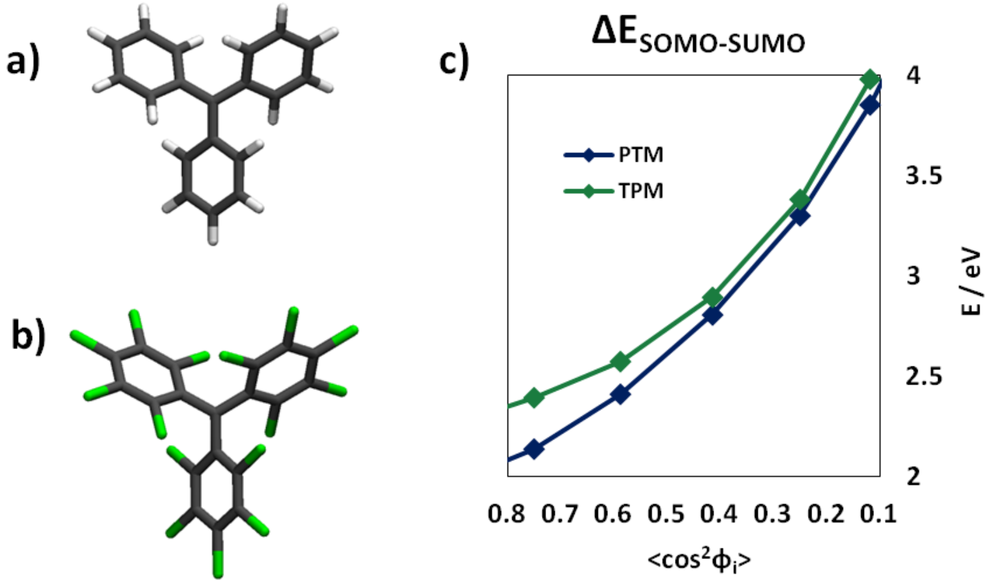

Fig. 5 The three aryl rings of the triphenylmethyl (TPM), (**a**) and perchloro-triarylmethyl (PTM), (**b**) TAM derivatives were systematically twisted from 20 to 90 degrees while relaxing the rest of the molecular structure by constrained optimizations. These calculations were done using the PBE0 hybrid functional[1] and the 6-311++g(d,p) basis set as implemented in the GAUSSIAN09[2] code. **c**) Resulting energy difference between the singly occupied (SOMO) and singly unoccupied (SUMO) molecular orbitals of the TPM (blue) and PTM (green) molecules against the corresponding $<\cos^2\phi_i>$ value (where $<\cos^2\phi_i> = (\cos^2\phi_1 + \cos^2\phi_2 + \cos^2\phi_3)/3$) during the constrained optimizations.

6. Magnetic coupling calculations

The description of the magnetic properties is based on the Ising spin Hamiltonian[5]

$$\hat{H}^{Ising} = -\sum_{\langle i,j \rangle} J_{ij} \hat{S}_i^z \hat{S}_j^z \qquad (1)$$

	2D-TPM					
	relaxed		Semi-distorted		distorted	
	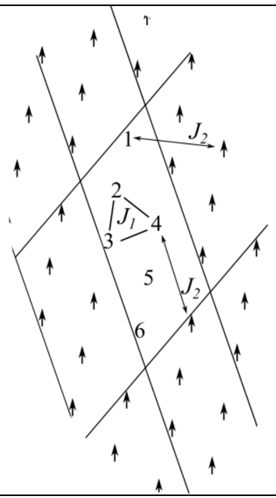		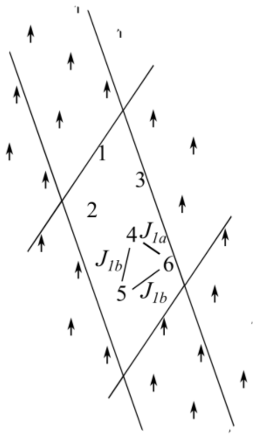		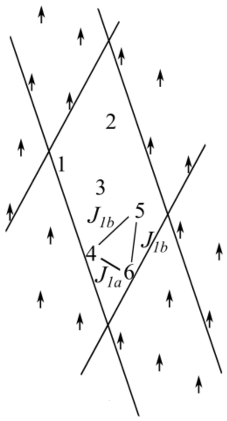	
solution	$FM = 123456$<br>$AFM_1 = 1\bar{2}3456$<br>$AFM_2 = 1\bar{2}34\bar{5}6$		$FM = 123456$<br>$AFM_1 = 12345\bar{6}$<br>$AFM_2 = 1\bar{2}3\bar{4}5\bar{6}$		$FM = 123456$<br>$AFM_1 = 1234\bar{5}6$<br>$AFM_1 = 1\bar{2}\bar{3}4\bar{5}6$	
	Energy expressions					
	E	ΔE_{FM-AFM}	E	ΔE_{FM-AFM}	E	ΔE_{FM-AFM}
FM	$-\frac{9}{2}(J_1 + J_2)$		$-\frac{1}{2}(3J_{1a} + 6J_{1b})$		$-\frac{1}{2}(3J_{1a} + 6J_{1b})$	
AFM_1	$-\frac{1}{2}(3J_1 + 5J_2)$	$-3J_1 - 2J_2$	$-\frac{1}{2}(J_{1a} + 2J_{1b})$	$-J_{1a} - 2J_{1b}$	$-\frac{1}{2}(J_{1a} + 2J_{1b})$	$-J_{1a} - 2J_{1b}$
AFM_2	$\frac{1}{2}(3J_1 - 9J_2)$	$-6J_1$	$\frac{1}{2}(-3J_{1a} + 6J_{1b})$	$-6J_{1b}$	$\frac{1}{4}(2J_{1a} + 5J_{1b})$	$-\frac{1}{4}(8J_{1a} + 17J_{1b})$
	Calculated energy differences per magnetic centre (cm^{-1})					
ΔE_{FM-AFM_1}	-16.0		-4.8		-0.1	
ΔE_{FM-AFM_2}	-32.1		-14.0		-0.3	
	Coupling constant values per magnetic centre (cm^{-1})					
J_{1a}	5.5		0.18		0.001	
J_{1b}	5.5		2.33		0.06	
J_2	-0.006		-		-	

Table 1 Schematic representation of the magnetic solutions used to extract the relevant coupling constants in each geometry of the 2D-TPM system, corresponding energy expressions as predicted by the Ising spin Hamiltonian, calculated energy differences and associated coupling constants values.

In order to extract all relevant magnetic interactions, it was necessary to define a magnetic cell which is double in one direction the unit cell. Thus, the magnetic cell shows six different radical centres which provide enough linearly independent equations to extract the coupling constants. The different magnetic solutions used are denoted FM, AFM1 and AFM2, as depicted in Table 1. With the energy expressions for each of the magnetic states obtained with Ising spin Hamiltonian, and the calculated energy differences associated with these states, based on the mapping approach[6] one can extract all relevant magnetic interactions.

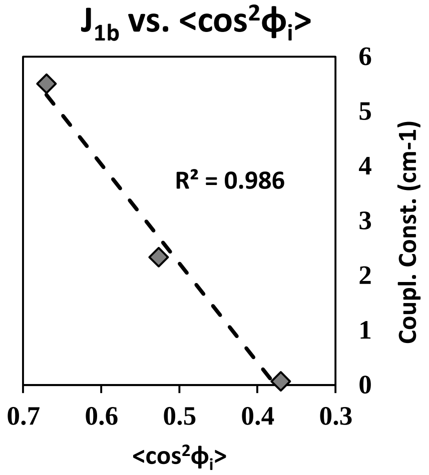

Fig. 6 Linear correlation between coupling constant values (cm^{-1}) against $<\cos^2\phi_i>$ for the different geometries (relaxed, semi-stretched, totally stretched) of the TPM 2D-COF. All values are calculated using the B3LYP[7] functional as implemented in the Crystal09 program.[8,9]

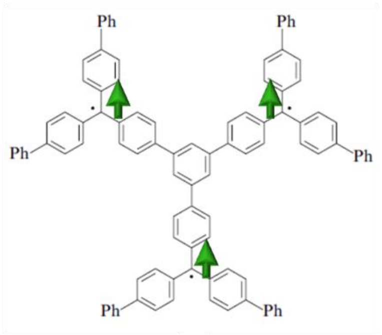

T = 93 K°

Fig. 7 Molecular structure of a tris-triphenylmethyl derivative experimentally synthesized and measured to present a quartet state at 93K° in solution.[4]

References

(1) Improta, R.; Barone, V. *Chem. Rev.* **2004**, *104* (3), 1231.

(2) Frisch, M. J.; Trucks, G. W.; Schlegel, H. B.; Scuseria, G. E.; Robb, M. A.; Cheeseman, J. R.; Scalmani, G.; Barone, V.; Mennucci, B.; Petersson, G. A.; Nakatsuji, H.; Caricato, M.; Li, X.; Hratchian, H. P.; Izmaylov, A. F.; Bloino, J.; Zheng, G.; Sonnenberg, J. L.; Had, M.; Fox, D. J. Gaussian, Inc.: Wallingford CT 2009,.

(3) Blum, V.; Gehrke, R.; Hanke, F.; Havu, P.; Havu, V.; Ren, X.; Reuter, K.; Scheffler, M. *Comp. Phys. Comm.* **2009**, *180* (11), 2175.

(4) Kothe, G.; Zimmermann, H. *Tetrahedron* **1973**, *29* (15), 2305.

(5) Ising, E. *Zeitschrift für Phys.* **1925**, *31* (1), 253–258.

(6) Moreira, I. de P. R.; Illas, F. *Phys. Chem. Chem. Phys.* **2006**, *8* (14), 1645–1659.

(7) Becke, a D. *J Chem Phys* **1993**, *103* (3-4), 361–363.

(8) Dovesi, R.; Orlando, R.; Civalleri, B. *Z. Krist.* **2005**, *220*, 571–573.

(9) Dovesi, R.; Saunders, V. R.; Roetti, C.; Orlando, R.; Zicovich-Wilson, C. M.; Pascale, F.; Civalleri, B.; Doll, K.; Harrison, N. M.; Bush, I. J.; D'Arco, P.; Llunell, M. *University of Torino*. Torino 2009.
